# Acetate Shock Loads Enhance CO Uptake Rates of Anaerobic Microbiomes

**DOI:** 10.1111/1751-7915.70063

**Published:** 2024-12-09

**Authors:** Alberto Robazza, Ada Raya i Garcia, Flávio C. F. Baleeiro, Sabine Kleinsteuber, Anke Neumann

**Affiliations:** ^1^ Institute of Process Engineering in Life Sciences 2: Electro Biotechnology Karlsruhe Institute of Technology – KIT Karlsruhe Germany; ^2^ Department of Microbial Biotechnology Helmholtz Centre for Environmental Research – UFZ Leipzig Germany

**Keywords:** acetic acid, acetogenesis, anaerobic digestion, hydrogenogenesis, methanogenesis, open mixed cultures, syngas, syntrophic acetate oxidation

## Abstract

Pyrolysis of lignocellulosic biomass commonly produces syngas, a mixture of gases such as CO, CO_2_ and H_2_, as well as an aqueous solution generally rich in organic acids such as acetate. In this study, we evaluated the impact of increasing acetate shock loads during syngas co‐fermentation with anaerobic microbiomes at different pH levels (6.7 and 5.5) and temperatures (37°C and 55°C) by assessing substrates consumption, metabolites production and microbial community composition. The anaerobic microbiomes revealed to be remarkably resilient and were capable of converting syngas even at high acetate concentrations of up to 64 g/L and pH 5.5. Modifying process parameters and acetate loads resulted in a shift of the product spectrum and microbiota composition. Specifically, a pH of 6.7 promoted methanogens such as *Methanosarcina*, whereas lowering the pH to 5.5 with lower acetate loads promoted the enrichment of syntrophic acetate oxidisers such as *Syntrophaceticus*, alongside hydrogenotrophic methanogens. Increasing acetate loads intensified the toxicity of undissociated acetic acid, thereby inhibiting methanogenic activity. Under non‐methanogenic conditions, high acetate concentrations suppressed acetogenesis in favour of hydrogenogenesis and the production of various carboxylates, including valerate, with product profiles and production rates being contingent upon temperature. A possible candidate for valerate production was identified in *Oscillibacter*. Across all tested conditions, acetate supplementation provided additional carbon and energy to the mixed cultures and consistently increased carboxydotrophic conversion rates up to about 20‐fold observed at pH 5.5, 55°C and 48 g/L acetate compared to control experiments. Species of *Methanobacterium*, *Methanosarcina* and *Methanothermobacter* may have been involved in CO biomethanation. Under non‐methanogenic conditions, the bacterial species responsible for CO conversion remain unclear. These results offer promise for integrating process streams, such as syngas and wastewater, as substrates for mixed culture fermentation allowing for enhanced resource circularity, mitigation of environmental impacts and decreased dependence on fossil fuels.

## Graphical abstract

Pyrolysis of lignocellulosic biomass yields syngas and an acetate‐rich wastewater. This study examined high acetate levels during syngas co‐fermentation by anaerobic microbiomes at different pHs and temperatures. Microbiomes converted syngas at 64 g/L acetate and pH 5.5. Acetate supplementation increased CO conversion rates and altered product spectrum and microbiota composition.

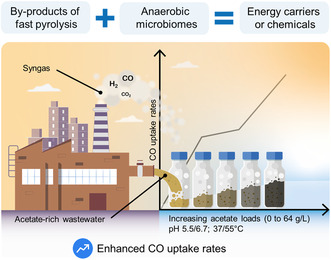

## 1 Introduction

Lignocellulosic biomass stands out as a primary renewable source of carbon and energy, offering a sustainable alternative to fossil‐derived materials (Rajesh Banu et al. 2021). However, due to the intricate polymeric structure and recalcitrant nature of the lignin within biomass, pretreatment technologies are essential to enhance conversion and recovery efficiencies (Velvizhi et al. 2022). Thermochemical processes are possible ways for the conversion of lignocellulose into compounds available for secondary processes. During pyrolysis, the cellulose and hemicellulose fractions undergo depolymerisation and deacetylation/cracking reactions producing acetate as by‐product (Sarchami, Batta, and Berruti 2021). The acetate contained in the pyrolysis vapours condenses through a series of condensation units, ultimately concentrating in an aqueous condensate. This condensate stands as wastewater of pyrolysis and is produced alongside with other products such as syngas (a mixture of gases including CO, CO_2_ and H_2_), biochar and bio‐oil.

To fully utilise all components of lignocellulosic biomass, it is crucial to recover secondary products generated during its thermochemical conversion, such as aqueous condensate and syngas and transform them into valuable products (Silva, Prunescu and Sin 2017). Biological processes offer a promising approach to valorize these residues thanks to their versatility and variety of products such as biofuels, biochemicals and bioplastics (Rajesh Banu et al. 2021; Velvizhi et al. 2022; Gonzales et al. 2016; Ruan et al. 2015). However, pyrolysis wastewater can contain toxic compounds such as phenols, furans, organic acids and heavy metals that inhibit microbial activity (Si et al. 2018). Anaerobic digestion stands out as a promising technology for wastewater valorization into biogas, owing also to its increased tolerance to toxicity compared to axenic processes (Feng and Lin 2017; Baêta et al. 2016). This process relies on the metabolic interplay of various microorganism groups to execute diverse parallel reactions with acetate as central metabolic intermediate (Pan et al. 2021; Batstone and Virdis 2014). Anaerobic cultures are pivotal for organic waste and wastewater management, enabling nutrient and energy recovery. Moreover, anaerobic microbiomes can exhibit tolerance to carbon monoxide toxicity in syngas. Microorganisms able to metabolise CO are also referred to as carboxydotrophs. Within anaerobic microbiomes, the products of their metabolism such as H_2_/CO_2_ or acetate can be directly utilised by methanogens to generate methane (Grimalt‐Alemany, Skiadas, and Gavala 2018; Zhang et al. 2021). The integration of anaerobic digestion of organic waste with syngas has been shown to significantly enhance methane production through syngas biomethanation (Postacchini et al. 2023; Luo, Wang, and Angelidaki 2013). Alternatively, in scenarios where methanogenesis is inhibited, anaerobic mixed cultures have demonstrated the capacity to accumulate medium‐chain carboxylates (MCCs) through a process known as chain elongation. The integration of syngas into chain elongation processes has shown promise in enhancing process efficiency by providing additional electron donors, which are crucial for the progressive reduction of short‐chain carboxylates (SCCs) into longer ones (Baleeiro, Kleinsteuber, and Sträuber 2021; Arslan et al. 2012). Moreover, the acetate produced from syngas fermentation by homoacetogens can undergo conversion in the presence of electron donors such as hydrogen, ethanol or lactate, contributing to the production of longer‐chain carboxylates (González‐Tenorio et al. 2020; Baleeiro et al. 2023a). The production of these compounds from waste streams is gaining interest due to their high value and their potential to serve as biofuel precursors, thereby mitigating reliance on fossil fuels (Baleeiro et al. 2019; Fuchs et al. 2023).

SCCs, such as acetate, serve as substrates for both methanogenesis and chain elongation processes, yet they can also exert inhibitory effects on microbial activity. Depending on pH, acetate exists in two forms: the dissociated form (CH₃COO^−^) and the undissociated form (acetic acid, CH₃COOH). In acidic environments (pH < 4.8), acetic acid is the dominant one, while at pH > 4.8, acetate mainly exists as the dissociated ion. These two forms of acetate influence microbial dynamics in distinct ways. The dissociated form has limited permeability across cell membranes, while the undissociated form can readily diffuse through them. Upon entering a more alkaline environment, such as the cellular cytoplasm, the undissociated form dissociates, releasing protons (H^+^). Cytoplasmic acidification can increase maintenance energy, disrupt enzymes activity and harm cellular components (Trček, Mira, and Jarboe 2015; Lawford and Rousseau 1994). High concentrations of undissociated acetate inhibit the growth of microbes, such as methanogens, reducing biogas production during anaerobic digestion processes or syngas biomethanation, for instance (Zhang et al. 2018; Han et al. 2019). Consequently, the loading of acetate‐rich wastewaters and process operations emerge as a crucial factors influencing microbial pathways and potential inhibition during syngas fermentation processes with anaerobic mixed cultures.

Despite the significance of this factor, our understanding of the co‐fermentation dynamics of acetate‐rich wastewater and syngas by anaerobic mixed cultures is limited, particularly regarding how varying acetate loadings impact microbial metabolism and product profiles. In this study, acetate was chosen as a model compound representing pyrolysis aqueous condensate due to its dual role as substrate and inhibitor in anaerobic digestion processes. The investigation focused on assessing the effects of increasing shock loads of acetate during syngas co‐fermentation with unacclimated anaerobic mixed cultures. Batch bottle experiments were conducted at different pH levels (6.7 and 5.5) and temperatures (37°C and 55°C) to evaluate metabolite production and microbial community composition.

## 2 Materials and Methods

### 2.1 Experimental Set‐Up, Fermentation Conditions and Community Analysis

Triplicates of each experimental condition (detailed in Table 1) were conducted in 250 mL serum bottles, each containing 50 mL of active volume, over a fermentation period of 16 days. The fermentation broth comprised 5 mL of sludge (10 v/v %), 5 mL of basal anaerobic (BA) medium (refer to Supporting Information for the composition), acetate (glacial acetic acid), 4 M NaOH for pH adjustment and deionised water to reach a final volume of 50 mL. All chemicals were purchased from Sigma‐Aldrich (Taufkirchen, Germany) or Carl Roth (Karlsruhe, Germany). The sludge utilised in the experiments was obtained from an anaerobic digester treating cow manure, later sieved to 0.5 mm and stored in the dark under anaerobic conditions at 4°C. Total suspended solids and volatile suspended solids were quantified at 41.6 ± 0.2 g/L and 17.7 ± 0.1 g/L, respectively, employing previously established methodologies (Telliard 2001). The experiments were performed over a period of 6 months and grouped in four sets. The consistency of the inoculum microbial community composition was monitored by collecting samples for amplicon sequencing every time it was used.

**TABLE 1:** Summary of the concentrations of acetate, acetic acid (HAc) and Na^+^ equivalents under the different experimental conditions. The term acetic acid used throughout this study refers only to the undissociated form of acetate. The term acetate, on the other hand, refers to the sum of both the dissociated and undissociated form of acetate.

Acetate [g/L]	pH 6.7			pH 5.5		
	Na^+^ eq. [g/L]	HAc [g/L]		Na^+^ eq. [g/L]	HAc [g/L]	
	37/55°C	37°C	55°C	37/55°C	37°C	55°C
0	0.85	0	0	0.22	0	0
1	1.21	0.01	0.01	0.51	0.16	0.17
2	1.58	0.02	0.02	0.85	0.31	0.33
4	2.5	0.05	0.05	1.49	0.63	0.67
6	3.6	0.07	0.07	2.13	0.94	1
8	3.97	0.09	0.1	2.87	1.25	1.33
12	7.1	0.14	0.15	3.79	1.88	2
16	7.83	0.19	0.2	4.89	2.51	2.67
24	11.69	0.28	0.3	7.28	3.76	4
32	15.37	0.37	0.4	9.67	5.02	5.34
40	18.5	0.46	0.5	11.73	6.27	6.67
48	22.17	0.56	0.6	14.62	7.53	8
56	24.38	0.65	0.7	16.75	8.78	9.34
64	29.16	0.74	0.8	20.33	10.04	10.67

Following the aliquoting of BA medium into each bottle, acetate was added and pH was adjusted to either 5.5 or 6.7. Deionised water was added to reach a volume of 44 mL. Subsequently, the serum bottles, deionised water and a 4 M NaOH solution were transferred into an anaerobic tent containing 5% H_2_ in N_2_ to anaerobize overnight. The bottles were inoculated with the anaerobic sludge, the pH levels were re‐adjusted to the desired values and any remaining volume was filled with anoxic deionised water. After sealing the bottles with rubber stopper and aluminium cap, the bottles underwent syngas flushing for 5 min, with a composition of 3 kPa H_2_, 20 kPa CO, 25 kPa CO_2_ and N_2_ at a total flow rate of 1 L/min, followed by pressurisation at room temperature up to a final absolute pressure of 210 kPa. The gas flow was controlled using high precision mass flow controllers from Vögtlin (Muttenz, Switzerland), while pressure in the bottles was monitored utilising a precision pressure indicator GMH 3100 Series (Greisinger, Mainz, Germany). Incubation was conducted at either 37°C or 55°C and 210 rpm in two Thermotron shaker incubators (Infors, Bottmingen, Switzerland). Three millilitres of the gas phase were sampled daily or depending on the rates of CO consumption. When the partial pressure of the system was found to be below 180 kPa or when the CO concentration in the gas phase was below 1%, the bottles were first flushed and then re‐pressurised according to the method described above. The molar concentrations of CO, CO_2_, H_2_, CH_4_ and N_2_ were determined using an Inficon 3000 Micro GC System equipped with a thermal conductivity detector, which employed a CP‐Molsieve 5 Å column and a PoraPLOT Q column at 80°C, with argon and helium serving as carrier gases, respectively. One millilitre of the fermentation broth was sampled every 2 days and centrifuged. The supernatant was filtered and stored at −20°C for later analytics. The concentrations of formate, acetate, ethanol, propionate, *n‐*butyrate and *n‐*valerate in the initial and final sample of each fermentation were measured by high‐performance liquid chromatography (HPLC) (Agilent 1100 Series, Agilent, Waldbronn, Germany). The HPLC was equipped with a Rezex ROA organic acid H+ (8%) column (300 × 7.8 mm, 8 μm; Phenomenex, Aschaffenburg, Germany) and a Rezex ROA organic acid H + (8%) guard column (50 by 7.8 mm) and run at 55°C with 5 mM H_2_SO_4_ at a flow of 0.6 mL/min. After the collection of the last sample, three 2 mL samples of the fermentation broth from bottles of some selected experiments (with 0, 6, 12, 40, 48, 56 and 64 g/L initial acetate at both pH and temperatures) were centrifuged for 15 min at 17,000 × *g*. The pellet was re‐suspended in 1 mL of phosphate‐buffered saline solution (pH 7.4) and underwent another round of centrifugation for 15 min at 17,000 × *g*. Upon removal of the supernatant, the pellets were stored at −20°C.

Amplicon sequencing of the 16S rRNA (V3‐V4 region) and *mcrA* genes was conducted using the Illumina MiSeq platform. Procedures for DNA extraction, PCR and library preparation were described previously (Logroño et al. 2020). The bioinformatics workflow for the visualisation of the microbial community composition and elaboration of Spearman correlations was performed as described in another work (Baleeiro, Kleinsteuber, and Sträuber 2022). The 16S rRNA‐ and *mcrA*‐based amplicon reads were rarefied to an equal sequencing depth of 17,000 and 1700 counts, respectively. Datasets with lower sequencing depth were excluded from the analysis. Consequently, at least duplicate datasets were kept from each experimental condition whereas under most experimental conditions triplicates were obtained. For conciseness, average abundance data of replicate communities are used to represent microbial community composition in this study. However, non‐metric multidimensional scaling (NMDS) plots were used as a visualisation aid for comparing the similarity between (1) samples of the inoculum throughout its storage time, (2) sample replicates and (3) samples under different experimental conditions. NMDS plots were elaborated using the phyloseq R package with Bray–Curtis dissimilarity (McMurdie and Holmes 2013).

### 2.2 Analytical Methods and Statistical Analysis

The total amount produced or consumed of each gas specimen was calculated via the ideal gas law as described by the following equation:

(1) $$n_{\mathrm{gas},i}=\sum_{t=0}^{j}\frac{p_{j}\times V_{j}}{R\times T}\;\left(\mathrm{mmol}\right),$$

where $n_{\mathrm{gas},i}$ is the cumulative consumption/production of a gas specimen *i*; $p_{j}$ [Pa] is the pressure of the head space of the bottle at sampling time; $V_{j}$ [m^3^] is the bottle's head space volume corrected for liquid sampling; R [j/mol/K] is the gas constant; T [K] is the incubation temperature; j is the number of samples.

Electron moles (e‐moles, e‐mol) were used to quantify the consumption and production flow of chemical compounds within the cultures as described in previous works (Esquivel‐Elizondo et al. 2017). The determination of the e‐moles space–time consumption/production rate for gases and metabolites was done following equation:

(2) $$q_{e-\mathrm{mol},i}=\frac{n_{i}\times {\mathrm{eeq}}_{i}}{V_{\text{Start}}\times t}\;\left(e-\mathrm{mM}/d\right),$$

where $n_{i}$ [mmol] is the absolute amount of each metabolite produced or consumed during the total fermentation time; ${\mathrm{eeq}}_{i}$ is the amount of the substance i that releases 1 e‐mol during complete oxidation (conversion tables are available in the Table S1); *V*_Start_ [L] is the volume of the fermentation broth at the start of the fermentation; and t [d] is the total fermentation time.

The product yields were calculated as described in the following equation. Hydrogen gas and acetate are regarded as substrates only when consumed, otherwise as products,

(3) $$\text{Product yield}=\frac{\sum n_{i}\times {\mathrm{eeq}}_{i}}{n_{\text{Syngas},\text{consumed}}\times {\mathrm{eeq}}_{\text{syngas}}+n_{\text{Acetate},\text{consumed}}\times {\mathrm{eeq}}_{\text{Acetate}}}\times 100\;\left(\%\right).$$

Calculations were conducted individually for each bottle and the results were averaged across the replicates (*n* = 3). The equations used to calculate C‐mol rates and C‐mol balances are available in the Supporting Information.

The term acetic acid (HAc) used throughout this study refers only to the undissociated form of acetate. Acetate, on the other hand, refers to the sum of both the dissociated and undissociated form of acetate. The concentration of HAc was calculated using a derivation of the Henderson‐Hasselbalch equation (Equation 4), as described in another work (Zhang et al. 2018):

(4) $$\mathrm{HAc}=\frac{C_{\text{Acetate}}\times C_{H^{+}}}{K_{a}+C_{H^{+}}}\times m\;\left(g/L\right),$$

where C_Acetate_ is the total concentration of acetate [mol/L], C_H_^+^ is the proton concentration [mol/L], m is the molecular mass of acetic acid [g/mol], and K_a_ is the dissociation constant [mol/L]. The K_a_ values for acetate were assumed to be 1.70∙10^−5^ [mol/L] and 1.58∙10–5 [mol/L] at 37°C and 55°C, respectively (Harned and Ehlers 1932).

## 3 Results and Discussion

### 3.1 Syngas and Acetate Metabolism of Anaerobic Mixed Cultures

**FIGURE 1:**
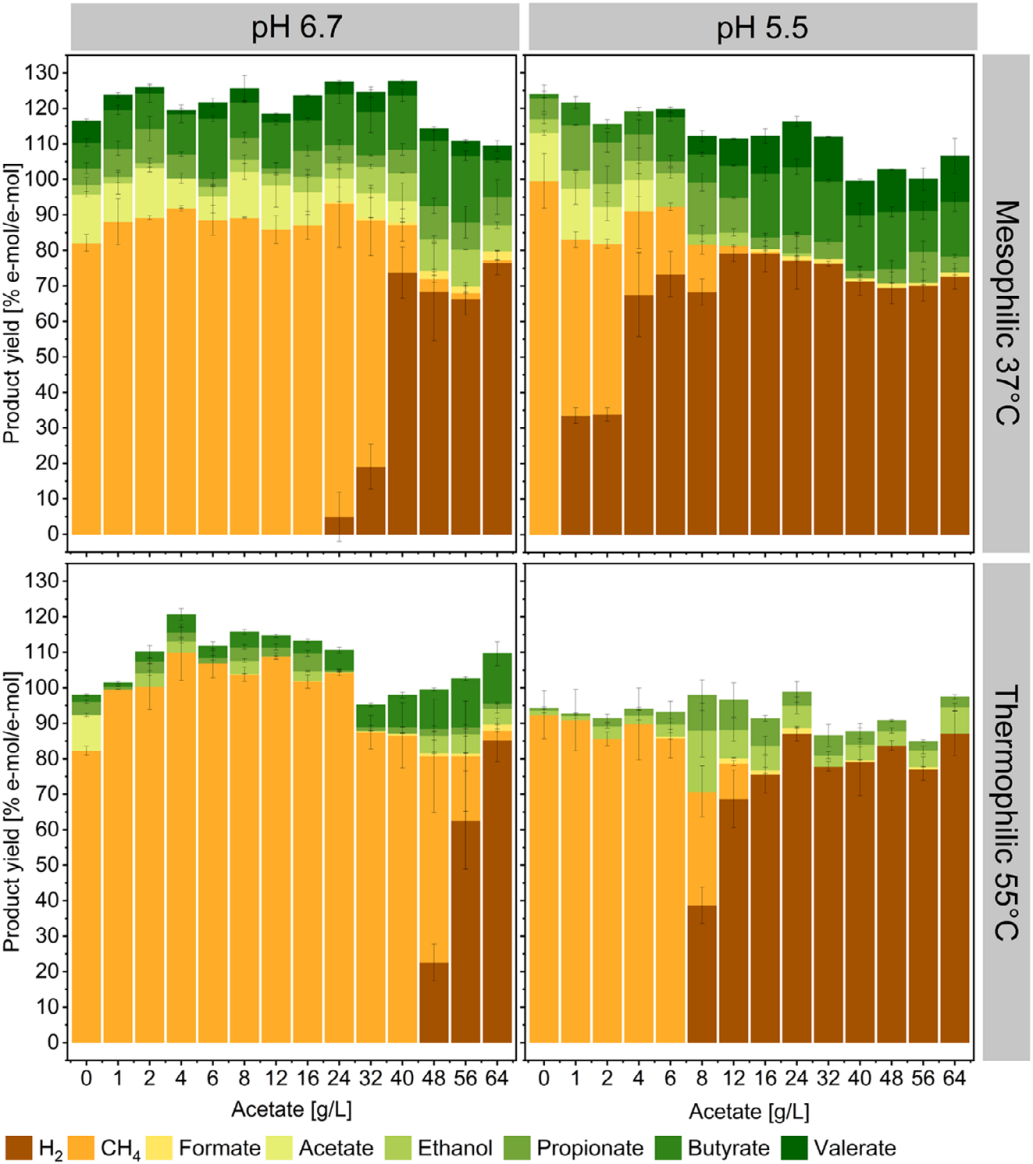
Electron mol balancing between substrates (CO; H_2_ and acetate if consumed) and products (CH_4_, formate, ethanol, propionate, butyrate and valerate; H_2_ and acetate if produced) at different process conditions and increasing acetate concentrations. Product recoveries higher that 100% may result from extra electron sources, such as solids in the inoculum. Error bars represent standard deviation among replicates (*n* = 3).

Syngas constituents and acetate were converted into methane, SCCs and ethanol or H_2_/CO_2_ depending on the environmental conditions and the thermodynamics of the various metabolic pathways involved. Product formation in terms of e‐mol yields is reported in Figure 1 (C‐mol yields are available in the Figure S1).

In general, mesophilic experiments resulted in high yields of methane, H_2_, SCCs and ethanol. Thermophilic conditions, on the other hand, decreased the production of SCCs and enhanced methanogenesis and hydrogenogenesis when compared to mesophilic experiments. Generally, methane is a primary metabolite in mesophilic and thermophilic anaerobic digester microbiomes (Angenent et al. 2016) and here, under low acetate concentrations and regardless of pH or temperature, over 80% of the electron equivalents from syngas and acetate were converted to methane. Other products in methanogenic processes are acetate, propionate and butyrate, especially at pH 37°C. The accumulation of SCCs during syngas methanation processes has been already reported and its extent depends also on the microbiota composition (Zhang et al. 2021; Grimalt‐Alemany et al. 2020a).

**FIGURE 2:**
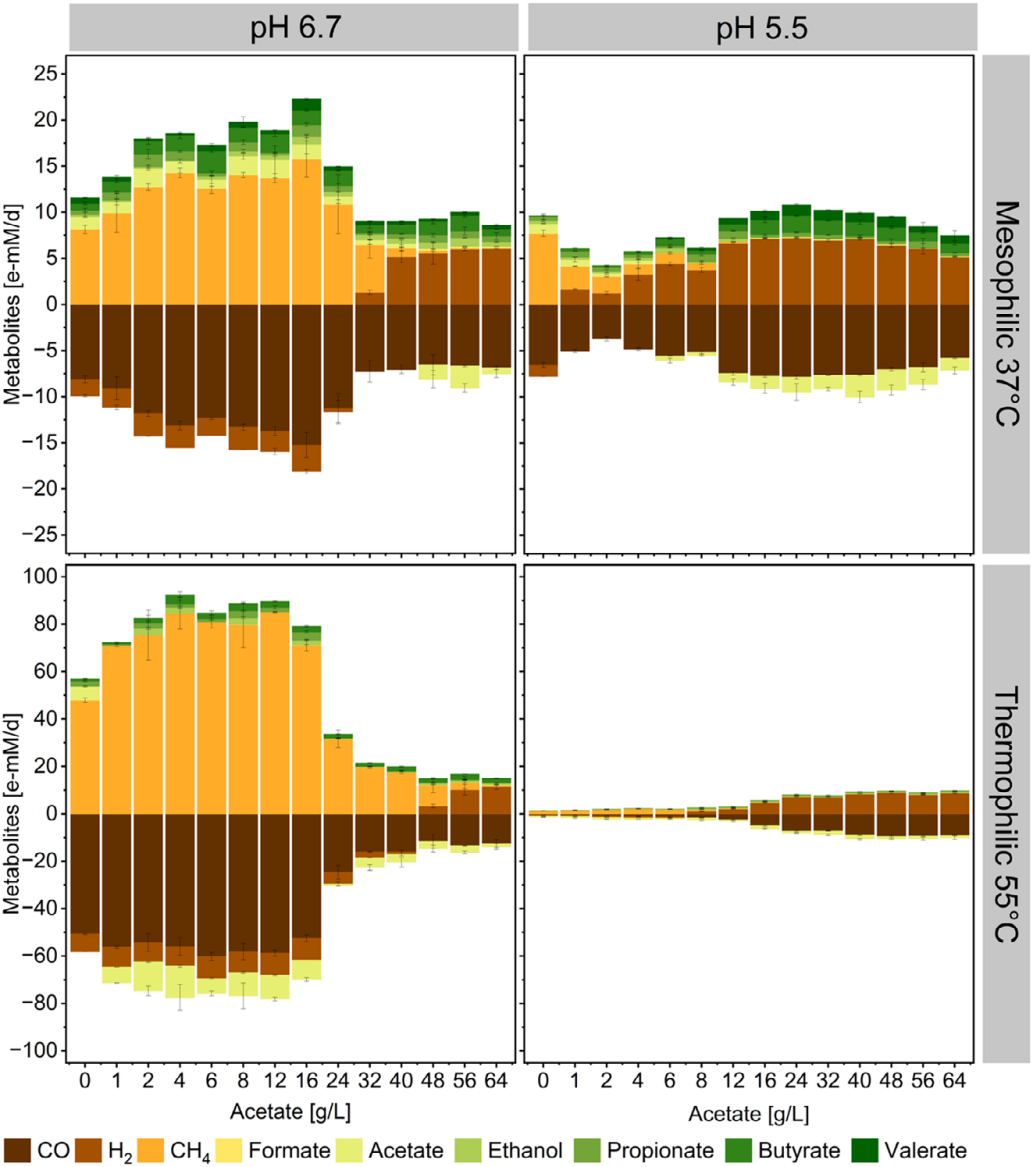
Consumption and formation rates of gaseous components (CO, H_2_ and CH_4_) and of some carboxylates (formate, acetate, propionate, butyrate and valerate) and ethanol at different process conditions and increasing acetate concentrations. Negative values indicate consumption. Error bars represent standard deviation among replicates (*n* = 3).

Mesophilic and thermophilic non‐methanogenic processes predominantly yielded hydrogen with an increased production of formate, ethanol, propionate, butyrate and valerate detected primarily at pH 6.7 or 37°C. The thermodynamics of CO‐consuming reactions at 55°C favour hydrogenogenesis over other carboxydotrophic reactions (Grimalt‐Alemany et al. 2020b), as detected here. At mesophilic conditions, on the other hand, homoacetogenesis should be the primary pathway of CO metabolism (Diender et al. 2015; Schoelmerich and Müller 2020). The dominance of hydrogenogenesis over homoacetogenesis in this work likely resulted from the elevated concentration of acetate, which may have inhibited acetate‐producing reactions, thereby prompting a shift in the microbial metabolism towards other products (el‐Gammal et al. 2017). A thermodynamic analysis determined at 310 K and pH 7 corroborates this hypothesis (Baleeiro et al. 2019). The Gibbs free energy of carboxydotrophic and hydrogenotrophic acetogenesis increases from −88 KJ/mol to −66.4 KJ/mol and −172 KJ/mol to −162 KJ/mol, respectively, when lowering the pH from 7 to 5.5 and in the presence of 100 mM acetate. Conversely, the Gibbs free energy of carboxydotrophic hydrogenogenesis, calculated under identical conditions, decreases from −20.9 to −24.1 KJ/mol. This may also explain the peaking acetate production rates detected at pH 6.7 and initial acetate concentrations up to 16 g/L (Figures 2 and S2), where the thermodynamic favourability of acetogenic reactions was possibly at its highest.

**FIGURE 3:**
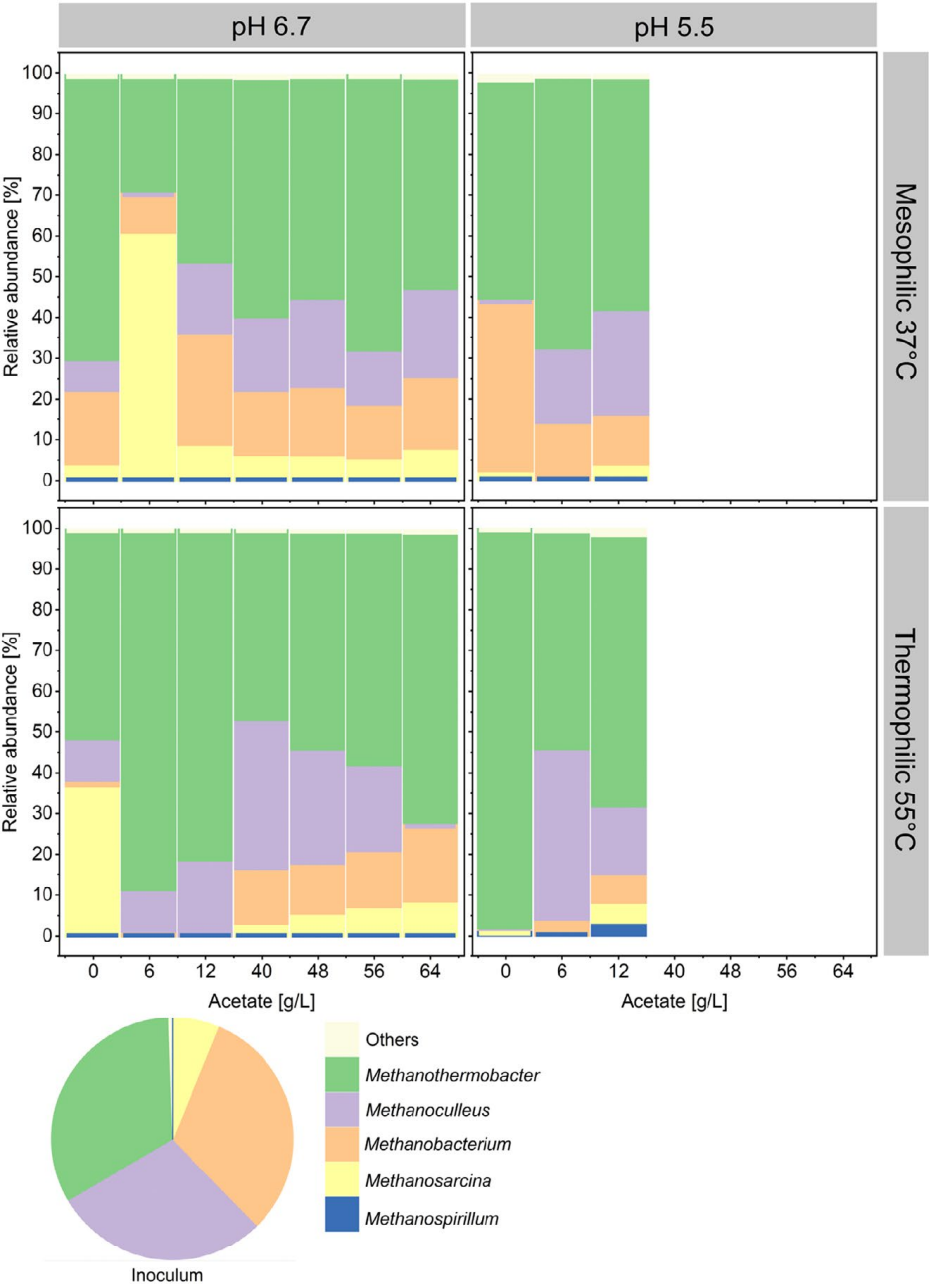
Average relative abundance of the enriched methanogenic genera (based on *mcrA* gene amplicon sequencing variants). Only the top five most abundant genera are shown. The rest are grouped in ‘Others’. Community analysis was performed only for cultures with methanogenic activity for the last samples collected at day 16. The microbial community composition for each replicate is available in the Figure S4.

A pH of 6.7 or a temperature of 55°C promoted acetate consumption. However, depending on the environmental conditions, two different pathways for acetate metabolism may have prevailed. In methanogenic processes with consumption of exogenous H_2_, acetoclastic methanogenesis or syntrophic acetate oxidation (SAO) likely served as the primary pathways for acetate consumption. Acetoclastic and hydrogenotrophic methanogenesis via SAO generally coexist during anaerobic digestion processes, with acetoclastic methanogenesis accounting for about 60%–70% of the total methane produced (Pan et al. 2021; Dyksma, Jansen, and Gallert 2020). The high relative abundance of *Methanosarcina* detected at 37°C, pH 6.7 (Figure 3) and low acetate concentrations may suggest the dominance of acetoclastic methanogenesis in those conditions. *Methanosarcina thermophila* ASV 008, for instance, was significantly correlated (*p* < 0.05) to increasing acetate concentration and pH 6.7 (Figure S3).

**FIGURE 4:**
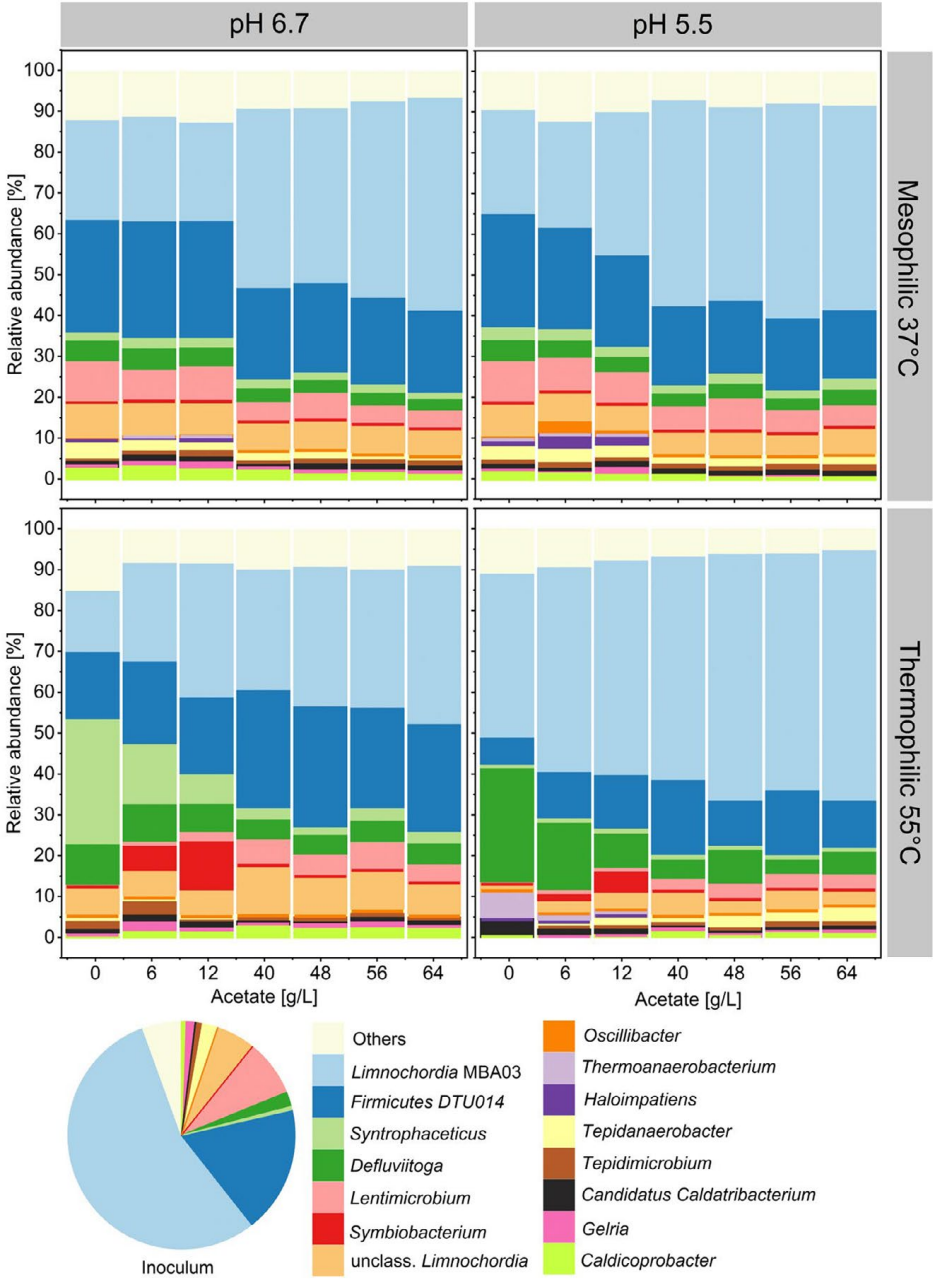
Average relative abundance of triplicate samples of the enriched bacterial genera (based on 16S rRNA amplicon sequencing variants). Only the top 15 most abundant genera are shown. The rest are grouped in ‘Others’. Community analysis was performed only for the last samples collected at day 16. The microbial community composition for each replicate is available in the Figure S5.

Although *Methanosarcina* species are versatile methanogens capable of acetoclastic, methylotrophic and hydrogenotrophic methanogenesis, they are often considered the primary acetoclastic methanogens during anaerobic digestion processes (De Vrieze et al. 2012). Some species of *Methanosarcina* such as *Methanosarcina barkeri* and *Methanosarcina acetivorans* have been reported to be able to consume CO for methanogenesis (Moran et al. 2008; O'Brien et al. 1984). Nevertheless, the presence of *Tepidanaerobacter*, a genus that includes syntrophic acetate oxidisers such as *Tepidanaerobacter acetatoxydans* (Müller et al. 2015), at relative abundances of up to 5% in mesophilic microbiomes under both pH conditions and initial acetate concentrations up to 12 g/L, suggests that SAO may still occur in mesophilic conditions (Figure 4).

Thermophilic conditions, on the other hand, may have promoted the development of syntrophic associations between hydrogenotrophic methanogens and syntrophic acetate oxidising bacteria. Hydrogenotrophic methanogenesis via SAO is generally the preferable route to methane production in anaerobic digestion at high temperatures, or in the presence of high ammonia or SCCs concentrations (Pan et al. 2021; Dyksma, Jansen, and Gallert 2020). SAO occurs only at low H_2_ partial pressure in association with H_2_‐consuming reactions such as hydrogenotrophic methanogenesis (Pan et al. 2021; Hattori 2008). In mesophilic processes (37°C), the overall reaction (SAO coupled to hydrogenotrophic methanogenesis) is exergonic only between 0.8 to 18 Pa H_2_, while at 55°C, the exergonic window ranges from 1.5 to 39 Pa H_2_ (Hattori et al. 2001; Schnürer, Svensson, and Schink 1997). Experimental studies reported that the H_2_ partial pressure during SAO ranged from 1.6 to 6.8 Pa in mesophilic processes (Schnürer, Svensson, and Schink 1997) and from 20 to 40 Pa in thermophilic ones (Hattori 2008; Hattori et al. 2001). Here, in most methanogenic processes with consumption of exogenous H_2_, H_2_ partial pressures at sampling time were 0. The average H_2_ partial pressures are shown in the Figure S6.

*Syntrophaceticus*, a genus known to harbour syntrophic acetate oxidisers (Westerholm, Dolfing, and Schnürer 2019), was enriched in thermophilic processes at pH 6.7 and acetate concentrations up to 12 g/L (Figure 4), confirming the favourability of these environments for SAO. *Syntrophaceticus* sp. ASV 011 was significantly correlated to acetate consumption (*p* < 0.05), high temperatures (*p* < 0.05) and to the highest methanogenesis rates of this work (*p* < 0.05) (Figure S7). Given its high abundance in the inoculum (Figure 3), *Methanoculleus* may have been associating with *Syntrophaceticus* for SAO. Species belonging to *Methanoculleus* such as MAB1 (phylogenetically affiliated to *Methanoculleus bourgensis* ) were proven to be suitable methanogenic partners for mesophilic syntrophic acetate‐oxidising bacteria such as *Syntrophaceticus schinkii* (Westerholm, Roos, and Schnürer 2010). The highest observed acetotrophic and methanogenic conversion rates within this study was detected in thermophilic conditions at pH 6.7 and 4 g/L initial acetate, highlighting the importance of SAO as a thermophilic methanogenic pathway. At 55°C and initial acetate concentrations lower than 16 g/L, *Methanothermobacter*, a hydrogenotrophic methanogen, was enriched to up to 90% relative abundance within the total methanogenic community (Figure 3). Although there is no evidence that *Methanothermobacter* was related to SAO in this work, *Methanothermobacter thermoautrophicus* was isolated as a methanogenic partner in thermophilic SAO (Balk, Weijma, and Stams 2002). Additionally, *Methanothermobacter* species such as *Methanothermobacter marburgensis* and *M. thermoautotrophicus* are known carboxydotrophic methanogens and here *Methanothermobacter* may have contributed to convert CO to CH_4_.

Under non‐methanogenic conditions, acetate may have undergone reduction reactions contributing together with CO to the formation of longer‐chain carboxylates, especially at 37°C. The interplay of high acetate concentrations and elevated H_2_ partial pressures likely steered the metabolic pathways of the microbial communities towards the synthesis of more reduced compounds. Another work evaluated the effects of acetate loads comparable to this work (0–50 g/L acetate, pH 5.5) during the mesophilic H_2_ production from sucrose with a non‐methanogenic anaerobic microbiome. Increasing the acetate load reduced sucrose consumption and hydrogen production. However, similarly to the results obtained here, acetate loads of 35 and 50 g/L promoted acetate uptake and the production of SCCs and MCCs such as butyrate, valerate and caproate, and ethanol (Wang et al. 2008).

While the conversion of CO into even‐number carboxylates like butyrate can happen either through direct pathways (Worden et al. 1989; Jeong et al. 2015) or via chain elongation processes (He et al. 2018; Liu et al. 2020), the direct production of odd‐number carboxylates such as propionate or valerate from CO was not yet documented (Moreira et al. 2021). Other works report of propionate accumulating in the fermentation processes involving mixed cultures fed with CO (He et al. 2018; Liu et al. 2020, 2014; Moreira et al. 2021), but it is considered to originate from lactate through the acrylate pathway or from protein‐rich waste via deamination and oxidation reactions (Liu et al. 2016). The propionate detected in this work may have originated from cell lysates generated during the inoculation phase, and it was subsequently converted to valerate through the reverse β‐oxidation, as described to occur even within chain elongation reactors solely fed with CO (He et al. 2018; De Smit et al. 2019). Albeit not abundant, *Oscillibacter* may have contributed to the chain elongation in this work. *Oscillibacter* was previously associated to the production of MCCs in reactor microbiomes, some of which were fed with syngas (Baleeiro et al. 2023a, 2023b; Joshi et al. 2021). A study on kangaroos' gut microbiota identified *Oscillibacter* as H_2_/CO_2_ utilisers but there are no other works confirming *Oscillibacter* as hydrogenotrophic microorganisms (Godwin et al. 2014). The thermophilic genus *Defluviitoga*, here enriched predominantly at 55°C (Figure 4), comprises thermophilic chemo‐organotrophic fermenting bacteria capable of converting polysaccharides into ethanol, acetate, H_2_ and CO_2_ (Maus et al. 2016). Here, *Defluviitoga* may have thrived on the remaining solids from inoculation. The presence of *Defluviitoga* in the microbiome enriched during syngas biomethanation in a trickle‐bed reactor was previously justified as a scavenger of dead cells (Asimakopoulos et al. 2019). Similarly, *Symbiobacterium*, here enriched up to about 10% in thermophilic conditions and 12 g/L initial acetate, is a thermophilic syntrophic genus that requires the assistance of other microbes to supplement growth factors (Ueda et al. 2004). *Symbiobacterium* may have benefited from cell lysate or from the cooperation with other community members. Carbon dioxide was determined to be the critical factor for *Symbiobacterium thermophilum* to grow in single culture (Watsuji et al. 2006; Ueda and Beppu 2007) while other *Symbiobacterium* species can produce acetate, propionate, butyrate and valerate from tryptone and yeast extract (Shiratori‐Takano et al. 2014). Some other works reported *Symbiobacterium* to be involved in SAO (Liu and Conrad 2011, 2010).

Thermophilic non‐methanogenic microbiomes fed with syngas typically generate primarily H_2_/CO_2_ and traces of carboxylates, with high selectivity above 90% towards acetate (Shen et al. 2018). However, such a trend was not observed in this study. Although H_2_ was indeed the major product, butanol and ethanol emerged as the primary metabolites in fermentation broths alongside with acetate consumption. Increased acetate concentrations decreased the thermodynamic favourability of the reaction responsible for its formation and promoted to its conversion into more reduced compounds. Another work reported that the acetate produced (up to about 6 g/L) during the microbial electrosynthesis from CO_2_ with anaerobic mixed cultures at pH 4 and 50°C was converted into ethanol and butyrate. While ethanol was likely produced via solventogenesis, butyrate was considered the result of the chain elongation of ethanol and acetate (Rovira‐Alsina, Balaguer, and Puig 2021).

Under mesophilic conditions, despite the varying acetate concentrations, the microbiomes remained largely similar to each other and to the inoculum, as illustrated by NMDS plots based on *mcrA* and 16S rRNA gene amplicons (see Figures S8 and S9). An exception occurred at pH 6.7 with an initial acetate concentration of 6 g/L, where the microbiome exhibited a different structure dominated by *Methanosarcina*, as previously mentioned. In contrast, thermophilic microbiomes showed greater dissimilarity from the inoculum. This increased variation is likely due to the higher temperature, given that the inoculum was collected from a mesophilic digester. At high acetate concentrations (≥ 40 g/L), minimal DNA turnover for the methanogenic community was observed, as the community profile closely resembled that of the inoculum. Under thermophilic conditions or at pH 5.5, replicates exhibited high dissimilarities, likely due to increased stress conditions. This phenomenon aligns with the Anna Karenina principle of ecology, whereby communities under severe stress often develop differently (Zaneveld, McMinds, and Thurber 2017) and has been already reported in other works. A work evaluating the microbial community shifts in biogas reactors under ammonia and acetate stress, for instance, reports of a high dispersion of the community composition although the reactors were operated under equal conditions (Lv et al. 2019).

### 3.2 Effects of Acetate Supplementation on Carboxydotrophic Conversion Rates

**FIGURE 5:**
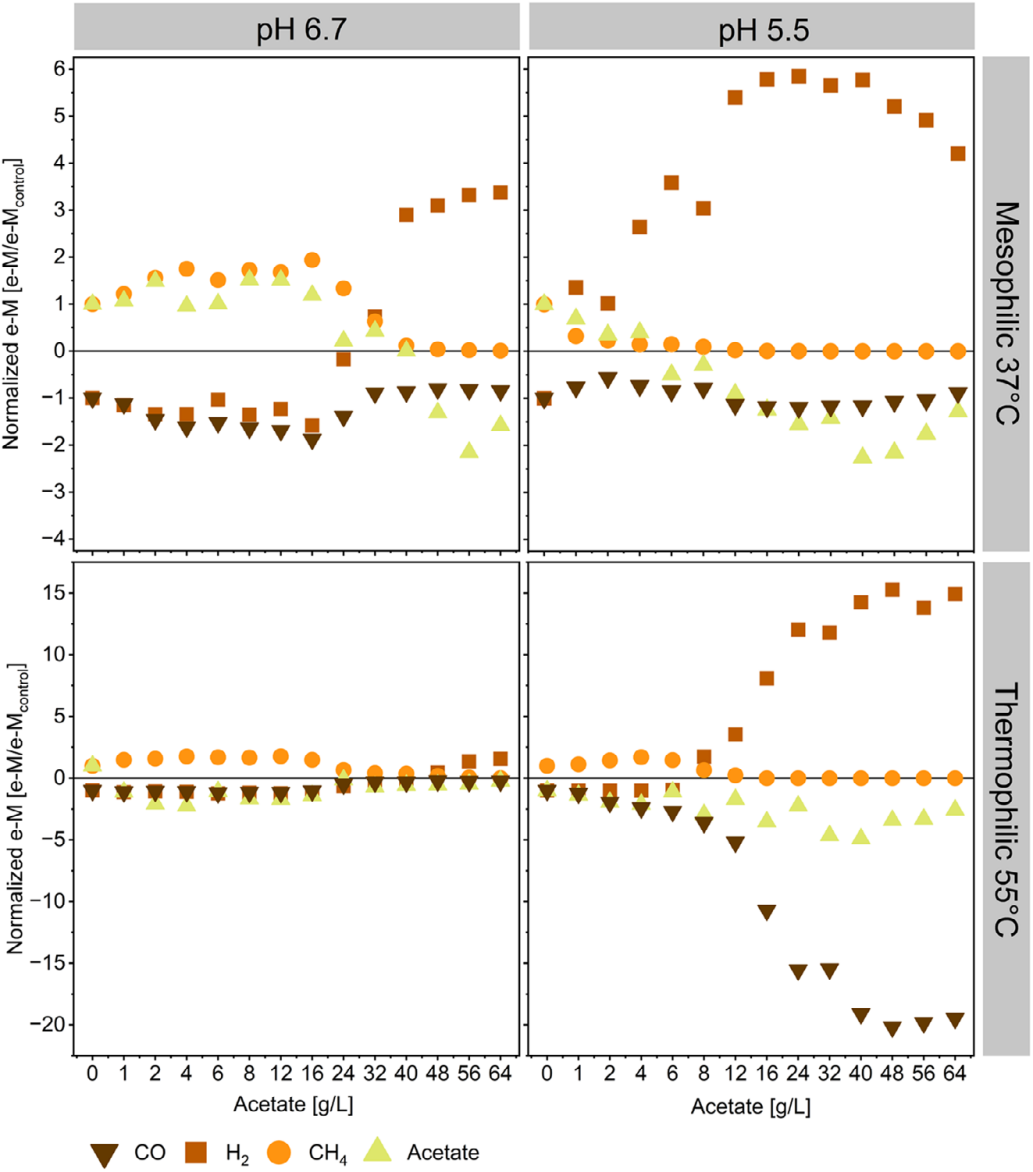
Cumulative e‐M for CO, H_2_, CH_4_ and acetate normalised to control experiments in the absence of supplemented acetate. Negative values indicate consumption while positive values production. Changes in sign (positive to negative, for instance) for a compound mark the switch of the metabolism compared to the control experiments.

Few of the genera enriched in this work have been reported to be carboxydotrophs or to have been enriched in processes with syngas as substrate. As mentioned before, *Methanothermobacter, Methanosarcina* and *Methanobacterium* are genera that comprise carboxydotrophic methanogens (Diender et al. 2015; Abbanat and Ferry 1990; Daniels et al. 1977). These genera were abundant in the inoculum and the presence of carboxydotrophic species and their potential involvement in CO uptake in methanogenic cultures cannot be excluded. *Thermoanaerobacterium*, here enriched to about 5% at 55°C and pH 5.5, was considered to be responsible for CO consumption during thermophilic syngas fermentation in hollow fibre bioreactors (Shen et al. 2018; Wang et al. 2017). It is plausible that the 16‐day incubation period was insufficient for the carboxydotrophic bacteria, assuming they contributed to CO consumption, to reach the top 15 most abundant genera. Multiple subculturing steps are typically necessary to enrich a defined and stable carboxydotrophic community in batch bottle experiments, a task not within the scope of this study. Nevertheless, in some conditions, increasing exogenous acetate supplementation enhanced carboxydotrophic conversion rates as depicted in Figure 5.

For instance, CO consumption peaked to 15.2 ± 1.3 e‐mM/d at 16 g/L initial acetate concentration, 37°C and pH of 6.7. A value nearly double the rate compared to control experiments in the absence of acetate (8.1 ± 0.4 e‐mM/day at 0 g/L acetate, 37°C and pH of 6.7) (Figure 5). At 55°C and pH 6.7, experiments supplemented with acetate showed a reduced lag phase and enhanced CO conversion rates throughout the whole fermentation period (the cumulative mmol for CO, H_2_ and CH_4_ over the fermentation time are available in the Figures S10–S12). An initial acetate concentration of 12 g/L (at 55°C and pH 6.7) resulted in a 16% increase in CO uptake rates. However, the kinetics of these experiments were constrained by experimental design, as syngas feeding was limited to once a day.

At 37°C and pH 5.5, carboxydotrophic conversion rates rose by 20% with initial acetate concentrations ranging from 16 to 24 g/L (equivalent to 2.5 and 5 gHAc/L) compared to control experiments. All acetate‐supplemented bottles at pH 5.5 and 55°C exhibited at least a 24% increase in CO uptake rates compared to experiments without acetate supplementation. There, carboxydotrophic conversion rates peaked at 20.1 ± 1.3 e‐mM/day, marking a 20‐fold increase compared to controls with 0 g/L acetate, when the initial acetate concentration reached 48 g/L (8 gHAc/L) (Figure 5).

Literature evaluating syngas fermentation at acetate concentrations comparable to this study is scarce. The highest recorded acetate concentrations produced from syngas have been achieved with *Acetobacterium woodii* (Kantzow, Mayer, and Weuster‐Botz 2015; Demler and Weuster‐Botz 2011; Straub et al. 2014). One work reported that 59.2 g/L acetate accumulated in the broth after 3 days of batch fermentation at pH 7. However, H_2_/CO_2_ uptake rates peaked at about 20 g/L (Kantzow, Mayer, and Weuster‐Botz 2015). Such high concentrations were achievable only because of the high pH, reducing the presence of HAc in the medium (Demler and Weuster‐Botz 2011). Nevertheless, some studies have highlighted the beneficial impact of acetate supplementation on single‐culture syngas fermentation. Although those studies used much lower acetate concentrations than the present work, they reported shorter lag phases, increased CO conversion rates, higher cell densities and improved growth rates. For instance, in batch fermentations with *Clostridium* sp. AWRP, 40 mM sodium acetate mitigated CO inhibition, eliminated the lag phase and increased CO consumption and production of ethanol and 2,3‐butanediol (Kwon, Lee, and Lee 2022). Similarly, adding 30 mM acetate to *Eubacterium limosum* fermentations improved cells growth, CO uptake and butyrate production rates. The reduced ferredoxin generated from CO oxidation was possibly used for ATP synthesis, which in turn drove acetate assimilation (Park et al. 2017). Carboxydotrophic microorganisms utilise membrane‐bound enzyme complexes such as Rnf or Ech to transfer electrons from reduced ferredoxin, produced during CO oxidation, to NAD^+^, while concurrently establishing an ion (H^+^ or Na^+^) potential across the cytoplasmic membrane (Hess, Schuchmann, and Müller 2013; Kaster et al. 2011). This electrochemical potential is then utilised by an ATP synthase to drive ATP synthesis (Katsyv and Müller 2020; Buckel and Thauer 2013; Schmidt, Biegel, and Müller 2009). It is known that some acetogenic bacteria are able to perform either homoacetogenesis from H_2_/CO_2_ or SAO in co‐culture with *Methanobacterium*, depending on the H_2_ partial pressure (Lee and Zinder 1988; González‐Cabaleiro et al. 2013). Additionally, in acetogenic clostridia such as *Clostridium ljungdahlii* or *Clostridium autoethanogenum*, the acetate in the fermentation broth determines the thermodynamic driving force of the aldehyde:ferredoxin oxidoreductase. The aldehyde:ferredoxin oxidoreductase regulates the availability of oxidised ferredoxin necessary for CO oxidation during the reduction of acetate to ethanol. An unfavourable acetate to ethanol ratio triggered oscillatory CO uptake rates of *Cl. autoethanogenum*, while external acetate supplementation improved the ethanol production from CO of *Cl. ljungdahlii* (Mahamkali et al. 2020; Schulz, Molitor, and Angenent 2023).

Here, acetate served as additional substrate in the systems, supporting different trophic groups of microbes that were favoured by varying environmental conditions. In general, acetate may have been directly assimilated by carboxydotrophic microorganisms. Alternatively, carboxydotrophic microorganisms might have been indirectly advantaged by the availability of metabolic intermediates produced by the acetate metabolism of other trophic groups. Overall, two general trends could be observed: at pH 6.7 and initial acetate concentrations below 16 g/L, acetate or the H_2_ generated from SAO may have promoted the activity of carboxydotrophic microorganisms such as the methanogens *Methanothermobacter*, *Methanobacterium* or *Methanosarcina* possibly contributing to the enhanced CO uptake rates. At initial acetate concentrations higher than 16 g/L, these processes were possibly inhibited but this aspect will be discussed in the next section. At pH 5.5, on the other hand, the inhibition of methanogenesis coincided with the increase of CO consumption, hydrogenogenesis and carboxylate production rates. Another work focusing on continuous mixed culture syngas fermentation in psychrophilic conditions reported of a 4.7‐fold increase of carboxydotrophic conversion rates as consequence of methanogenesis inhibition due to pH lowering (Andreides, Lopez Marin, and Zabranska 2024). Hydrogenotrophic methanogens generally outcompete homoacetogens for H_2_, especially at low H_2_ partial pressures like in this study (Weijma et al. 2002). However, methanogenesis inhibition may have allowed for the accumulation of H_2_ and formate, as mentioned before. Formate and H_2_ are the primary interspecies electron donors in anaerobic environments and are key metabolites in the Wood‐Ljungdahl pathway of acetogens (Takors et al. 2018; Schink 1997; Thiele and Zeikus 1988; Kouzuma, Kato, and Watanabe 2015). A higher H_2_ availability may have improved the redox state of the cells via the activity of hydrogenases or promoted acetate chain elongation into longer‐chain carboxylates (Lee et al. 2022; Valgepea et al. 2018). Formate was reported to increase CO tolerance of *A. woodi* and of chain‐elongating microbiomes via the formate dehydrogenase (Baleeiro et al. 2023b; Bertsch and Müller 2015; Ragsdale and Pierce 2008).

### 3.3 Inhibition of Methanogenic Activity

The inhibition of methanogenic activity likely resulted from the presence of HAc and Na^+^ in the fermentation media. Under conditions of salt or acid stress, cells typically expel potassium ions to counteract the levels of intracellular Na^+^ ions or HAc. This process places an additional energy burden on the cells, diverting ATP resources towards maintaining pH homeostasis rather than anabolic reactions, thus lowering microbial growth (Oren 2011). At pH 6.7, severe inhibition of methanogenesis was observed at approximately 18.4 gNa^+^/L (40 g/L acetate or 0.46 gHAc/L) and 37°C, while at thermophilic conditions, achieving similar inhibition levels required 24 g/L Na^+^ (56 g/L acetate or 0.7 gHAc/L) (Table and Figure 1). At pH 5.5, on the other hand, methanogenesis was completely inhibited by 2 gHAc/L (3.7 gNa^+^/L and 12 g/L initial acetate) for both mesophilic and thermophilic cultures. Experiments at pH 5.5 and 55°C showed very low microbial activity possibly as consequence of the pH and temperature shocks after inoculation, extending the lag phase. The IC50 values (i.e., inhibitor concentrations causing a 50% decrease in microbial activity) of Na^+^ for anaerobic digestion reported in the literature range from 3 to 53 gNa^+^/L (Chen, Cheng, and Creamer 2008; Feijoo et al. 1995). Sodium chloride concentrations higher than 9 g/L increased the lag phase and decreased both the growth rates and CO conversion rates of *Clostridium carboxidivorans* , while 18 g/L NaCl (about 6.8 gNa^+^/L) caused complete inhibition (Fernández‐Naveira, Veiga, and Kennes 2019). Six grams per litre of sodium ions inhibited the microbial electrosynthesis of acetate from bicarbonate via anaerobic mixed cultures (Dessì et al. 2023). Additionally, 8 gNa^+^/L inhibited hydrogen production by 50% during dark fermentation with anaerobic mixed cultures (Lee et al. 2012). The high variability of performances of processes under sodium stress depends on several factors such as reactor microbiota, process design, substrate and presence of other cations (Hierholtzer and Akunna 2012). Here, Na^+^ inhibitory concentrations were in the range of 18–24 gNa^+^/L but the presence of HAc may have contributed to increasing process inhibition. HAc, on the other hand, was probably the primary inhibitor of methanogenesis at pH 5.5. This is consistent with findings from other studies where 2.3 gHAc/L resulted in at least 90% inhibition of methanogenesis (Zhang et al. 2018, 2013; Wang et al. 2017).

When comparing methanogenesis with carboxydotrophic activity, methanogenic reactions exhibited earlier signs of inhibition than carboxydotrophic reactions in response to both HAc and Na^+^. The tolerance to high salt or other ions concentrations depends on the amount of energy that can be conserved during the catabolic reactions and on the coping mechanisms employed by the various microorganisms (Oren 2011). In this work, the lower inhibition of carboxydotrophic reactions may suggest that they were among the most energetically favourable pathways. While some homoacetogens such as *A. woodii* require external supplementation of Na^+^ (about 0.7 g/L) during growth on H_2_/CO_2_, others were reported to survive in acidic and alkaline environments or to have a higher upper salt limit than methanogens (Oren 2011, 1999; Muller et al. 1990; Reno, Volker, and Gerhard 1989; Drake, Gößner, and Daniel 2008). *Cl. ljungdahlii* was reported to form biofilms as stress response to 3.5 gNa^+^/L while growing on 200 mM fructose (Philips et al. 2017). The formation of granules or biofilms increases stress resistance and facilitates microbial interactions (Philipp et al. 2023), but no evidence about their formation was found here. Nonetheless, signs of inhibition in mesophilic and thermophilic experiments were evident also for CO conversion at pH 6.7 and high acetate concentrations. Initial acetate concentrations of 40 g/L (about 18 gNa^+^/L) inhibited mesophilic and thermophilic CO uptake rates by about 20% and 70%, respectively.

## 4 Conclusions

Recovering energy from lignocellulosic biomass is essential for reducing reliance on fossil fuels, enhancing resource circularity and mitigating environmental impacts. This study elucidates the potential of utilising acetate‐rich wastewater as a co‐substrate in batch syngas fermentation with mesophilic and thermophilic microbiomes under varying pH levels (6.7 and 5.5).

Microbiomes proved to be able to perform syngas fermentation even at high acetate concentrations and acidic pH levels. The microbial diversity of mixed cultures allowed for a highly flexible and resilient metabolism capable of adapting to changing environmental conditions. Manipulating process conditions and acetate loads allowed for steering the metabolism of the mixed culture, promoting favourable reactions while inhibiting others. Specifically, pH 6.7 promoted methanogenic reactions, whereas lowering the pH to 5.5 intensified the toxicity of undissociated acetic acid, thereby inhibiting methanogenesis at lower acetate loads. Under non‐methanogenic conditions, acetate stimulated hydrogenogenesis and the production of various carboxylates, including valerate, contingent upon temperature. Acetate supplementation enhanced CO conversion rates by providing extra carbon and energy sources to the process.

The results obtained in this work may be relevant for technologies that aim to combine different waste streams. Future research should focus on assessing the feasibility of acetate and syngas co‐fermentation in continuous cultivation setups to elucidate process performance nuances and identify key carboxydotrophic microorganisms capable of thriving under high acetate loads.

## Author Contributions

**Alberto Robazza:** conceptualization, methodology, data curation, investigation, validation, formal analysis, visualization, resources, writing – original draft, writing – review and editing. **Ada Raya i Garcia:** data curation, investigation, formal analysis. **Flávio C. F. Baleeiro:** conceptualization, methodology, data curation, validation, visualization, resources, writing – review and editing, formal analysis. **Sabine Kleinsteuber:** conceptualization, resources, writing – review and editing, validation. **Anke Neumann:** conceptualization, writing – review and editing, resources, project administration, supervision, funding acquisition, validation.

## Conflicts of Interest

The authors declare no conflicts of interest.

## Supporting information


Data S1.

## Data Availability

All data generated or analysed during this study are included in this published article and its Supporting information files. The raw sequence data without adapters have been deposited in the European Nucleotide Archive (ENA) under the study accession number PRJEB77205.
