# Supplementary material for: Acetate Shock Loads Enhance CO Uptake Rates of Anaerobic Microbiomes
Source: Microb Biotechnol. 2024 Dec 9;17(12):e70063. doi: 10.1111/1751-7915.70063 (PMC11626651; doi:10.1111/1751-7915.70063)
Supplement: Supplementary file 1 — Data S1. [file MBT2-17-e70063-s001.docx]

Acetate shock loads enhance CO uptake rates of anaerobic microbiomes

Alberto Robazza^1^, Ada Raya i Garcia^1^, Flávio C. F. Baleeiro^2^, Sabine Kleinsteuber^2^; Anke Neumann^1^

^1^ Institute of Process Engineering in Life Sciences 2: Electro Biotechnology, Karlsruhe Institute of Technology - KIT, 76131 Karlsruhe, Germany;

^2^ Department of Microbial Biotechnology, Helmholtz Centre for Environmental Research – UFZ, 04318 Leipzig, Germany

# Fermentation medium

For each liter of modified basal anaerobic medium were added: 100 mL of mineral salt solution, 800 mL of phosphate buffer solution, 10 mL of vitamin solution, 10 mL of trace elements solution, 5 mL of resazurin solution and 3 mL of reducing agent solution.

The mineral salt solution was prepared with the following salt concentrations: NH_4_Cl, 161.2 g/L; MgCl_2_ × 6H_2_O, 5.4 g/L; CaCl_2_ × 2H_2_O 6.5 g/L, NaCl, 30 g/L.

The phosphate buffer solution was prepared with 136 g/L KH_2_PO_4_.

The vitamin solution was composed of: biotin, 0.002 g/L; folic acid, 0.002 g/L; pyridoxin, 0.01 g/L; thiamin, 0.005 g/L; riboflavin, 0.005 g/L; nicotinic acid, 0.005 g/L; Ca-pantothenate, 0.005 g/L; vitamin B12, 0.005 g/L; aminobenzoic acid, 0.005 g/L; liponic acid, 0.005 g/L).

Trace elements solution contained the following compounds: FeCl_2_ × 4H_2_O, 1.5 g/L; MnCl_2_, 0.1 g/L; CoCl_2_ × 6H_2_O, 0.19 g/L; ZnCl2, 0.07 g/L; CuCl2 × 2H_2_O, 0.002 g/L; NiCl2 × 6H_2_O, 0.024 g/L; Na_2_MoO_4_ × 2H_2_O, 0.036 g/L; H_3_BO_3_, 0.006 g/L; Na_2_SeO_3_ × 5H_2_O, 0.003 g/L; Na_2_WO_4_ × 2H_2_O, 0.02 g/L.

The reducing agent solution contained 100 g/L of L-cysteine.

The resazurin solution contained 1 g/L resazurin sodium salt.

# Electron-mol balances

Table S1. Conversion factors for electron balances.

| Compound | Chemical Formula | Molecular Weight | mol e^-^ /mol |
| --- | --- | --- | --- |
| Formate | CH_2_O_2_ | 46.1 | 2.0 |
| Acetate | C_2_H_4_O_2_ | 60.0 | 8.0 |
| Ethanol | C_2_H_6_O | 46.0 | 12.0 |
| Propionate | C_3_H_6_O_2_ | 74.0 | 14.0 |
| Butyrate | C_4_H_8_O_2_ | 88.1 | 20.0 |
| Valerate | C_5_H_10_O_2_ | 102.1 | 26 |
| Hydrogen | H_2_ | 2.0 | 2.0 |
| Carbon Monoxide | CO | 28.0 | 2.0 |
| Carbon Dioxide | CO_2_ | 44.0 | 0.0 |
| Methane | CH_4_ | 16.0 | 8.0 |

# Equations

The determination of the C-moles space-time consumption/production rate for gases and metabolites as depicted in Figure S2 was performed following Eq.S1.

$q_{C-mol, i}=\frac{n_{i}*{C\mathrm{eq}}_{i}}{V_{\mathrm{Start}}*t}$[C-mM/d] Eq.S1

Where $n_{i}$ [mmol] is the absolute amount of each metabolite produced or consumed during the total fermentation time; ${C\mathrm{eq}}_{i}$ is the amount of carbon atoms contained in each compound; V_Start_ [L] is the volume of the fermentation broth at the start of the fermentation; $t$ [d] is the total fermentation time.

The product yields (based on C-mols) as shown in Figure S1 were calculated as described in Eq. S2. Acetate is regarded as substrates only when consumed, otherwise as products.

$Product yield=\frac{\sum n_{i}*{C\mathrm{eq}}_{i}}{n_{Syngas, consumed}*{C\mathrm{eq}}_{\mathrm{syngas}}+n_{Acetate,consumed}*{C\mathrm{eq}}_{\mathrm{Acetate}}}*100$ [%] Eq.S2

All calculations were conducted individually for each bottle and the results were averaged across the replicates (*n*=3).





Figure S1. Carbon balancing between substrates (CO; CO_2_ and acetate if consumed) and products (CH_4_, formate, ethanol, propionate, butyrate and valerate; CO_2_ and acetate if produced) at different process conditions and increasing acetate concentrations. Error bars represent standard deviation among replicates (n=3).





Figure S2. Consumption and formation rates of syngas components (CO, CO_2_ and CH_4_) and some short-chain carboxylates (formate, acetate, propionate, butyrate and valerate) and ethanol at different process conditions and increasing acetate concentrations. Negative values indicate consumption. Error bars represent standard deviation among replicates (n=3).


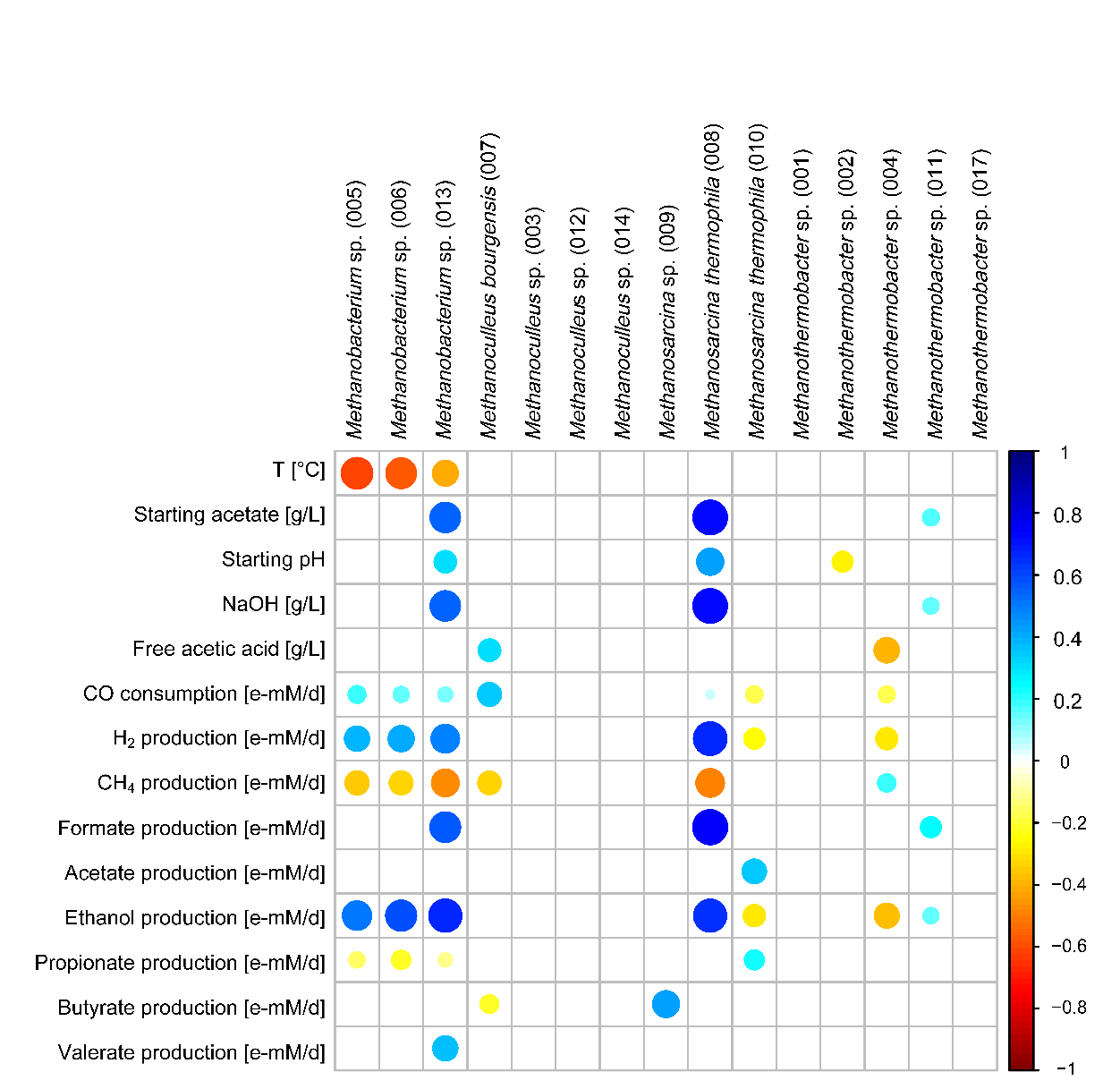


Figure S3. Spearman’s rank correlations (p<0.05) between relative abundance of dominant amplicon sequencing variants (ASVs) based on mcrA gene and process parameters. The strength of the correlation is represented by the size of the circle and intensity of the color. Blue circles indicate positive correlations. Red circles indicate negative correlations.


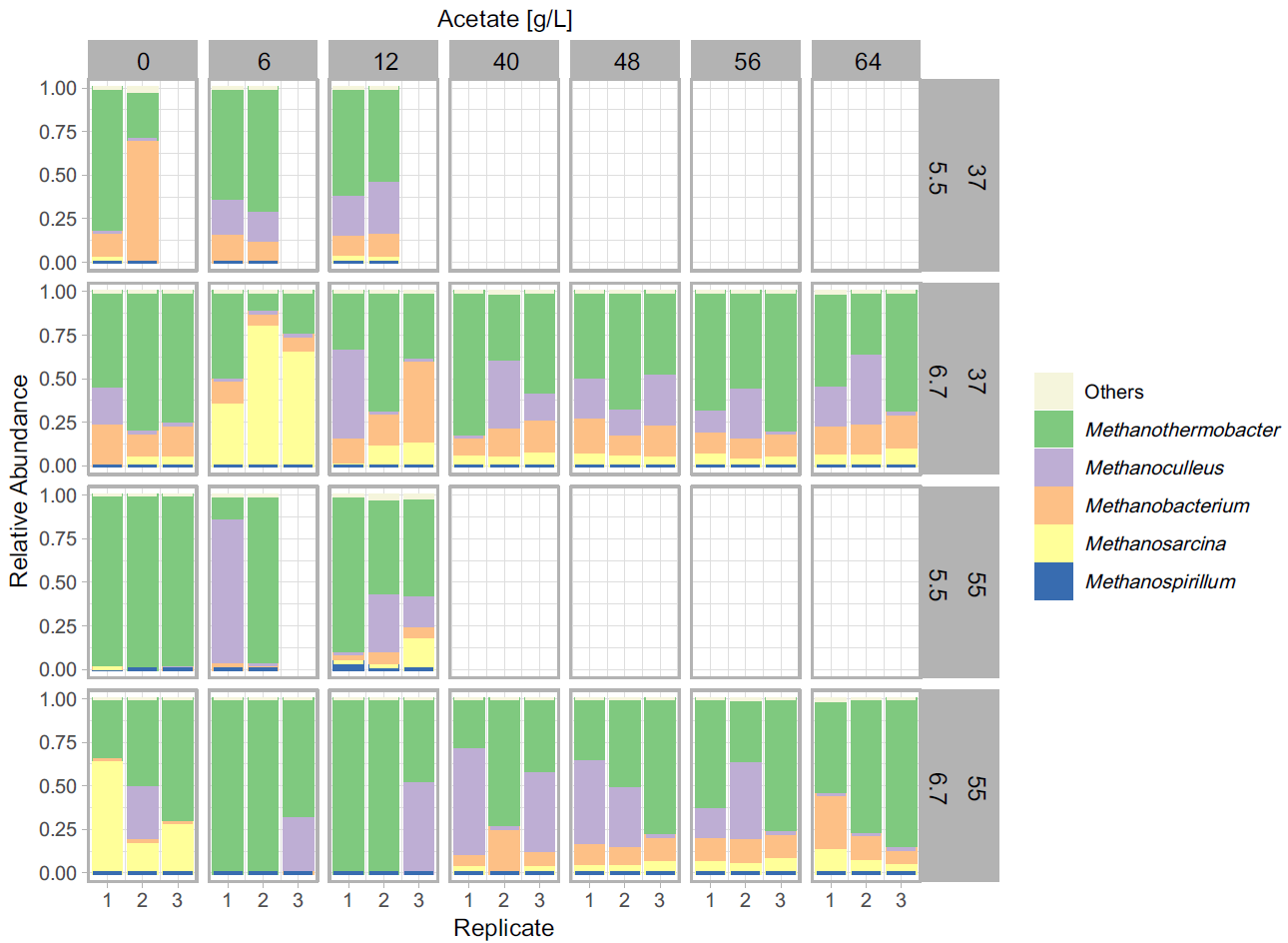
 Figure S4. Relative abundance of the enriched methanogenic genera (based on mcrA gene amplicon sequencing variants) for each replicate. Only the top 5 most abundant genera are shown. The rest are grouped in “Others”. Community analysis was performed only for cultures with methanogenic activity.


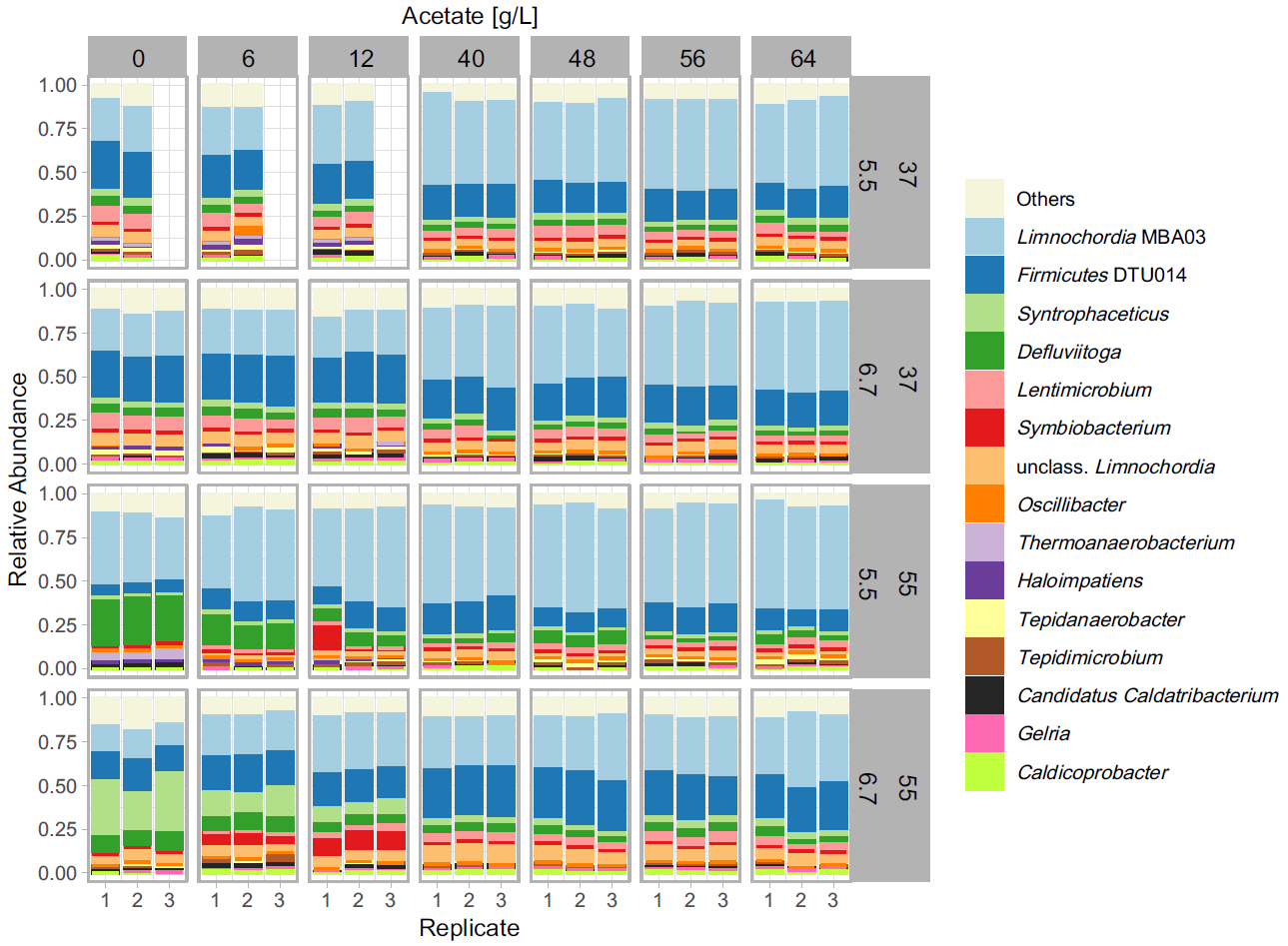
 Figure S5. Relative abundance of the enriched bacterial genera (based on 16S rRNA amplicon sequencing variants) for each replicate. Only the top 15 most abundant genera are shown. The rest are grouped in “Others”.





Figure S6. Average H_2_ partial pressure throughout the whole fermentation time. Values were determined from H_2_ concentrations in the headspace of the bottles at each sampling time. Error bars represent standard deviation among replicates (n=3).


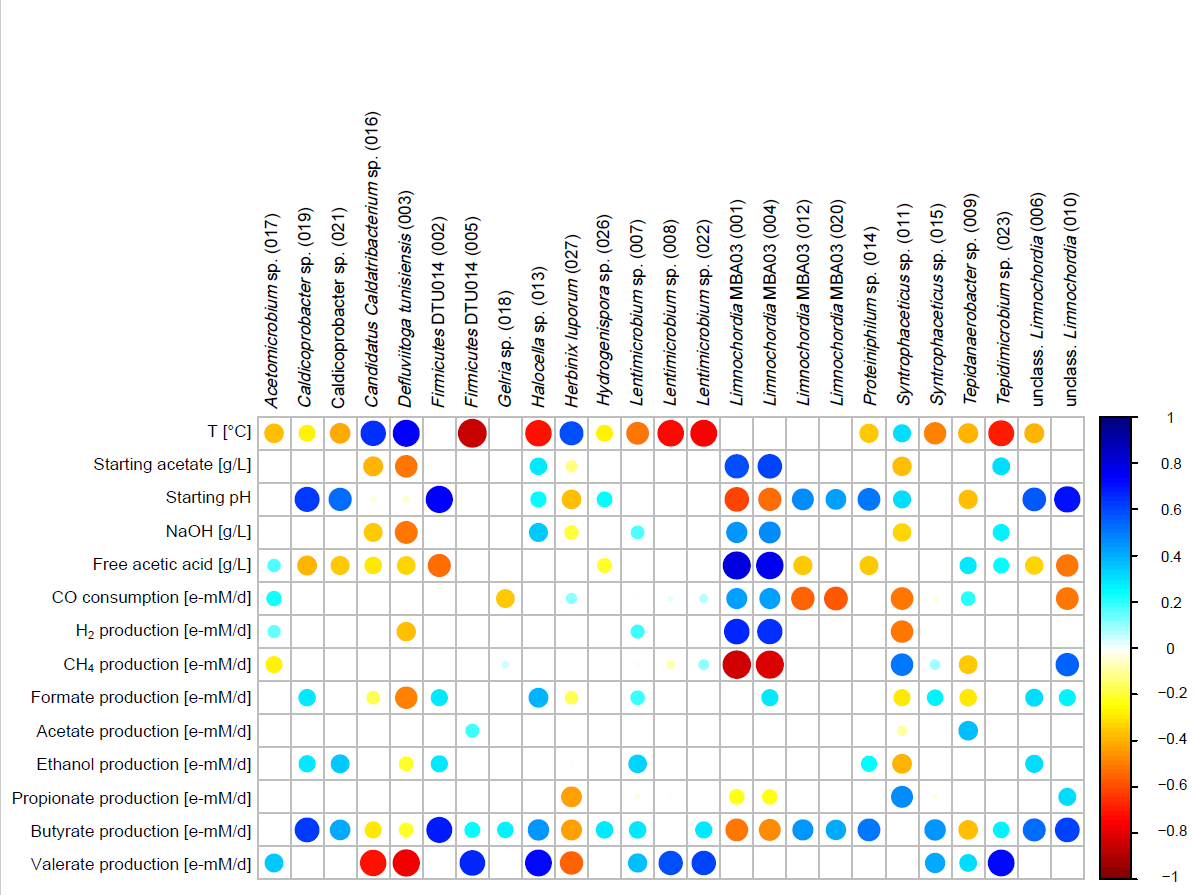


Figure S7. Spearman’s rank correlations (p<0.05) between relative abundance of dominant amplicon sequencing variants (ASVs) based on 16S rRNA gene and process parameters. The strength of the correlation is represented by the size of the circle and intensity of the color. Blue circles indicate positive correlations. Red circles indicate negative correlations.


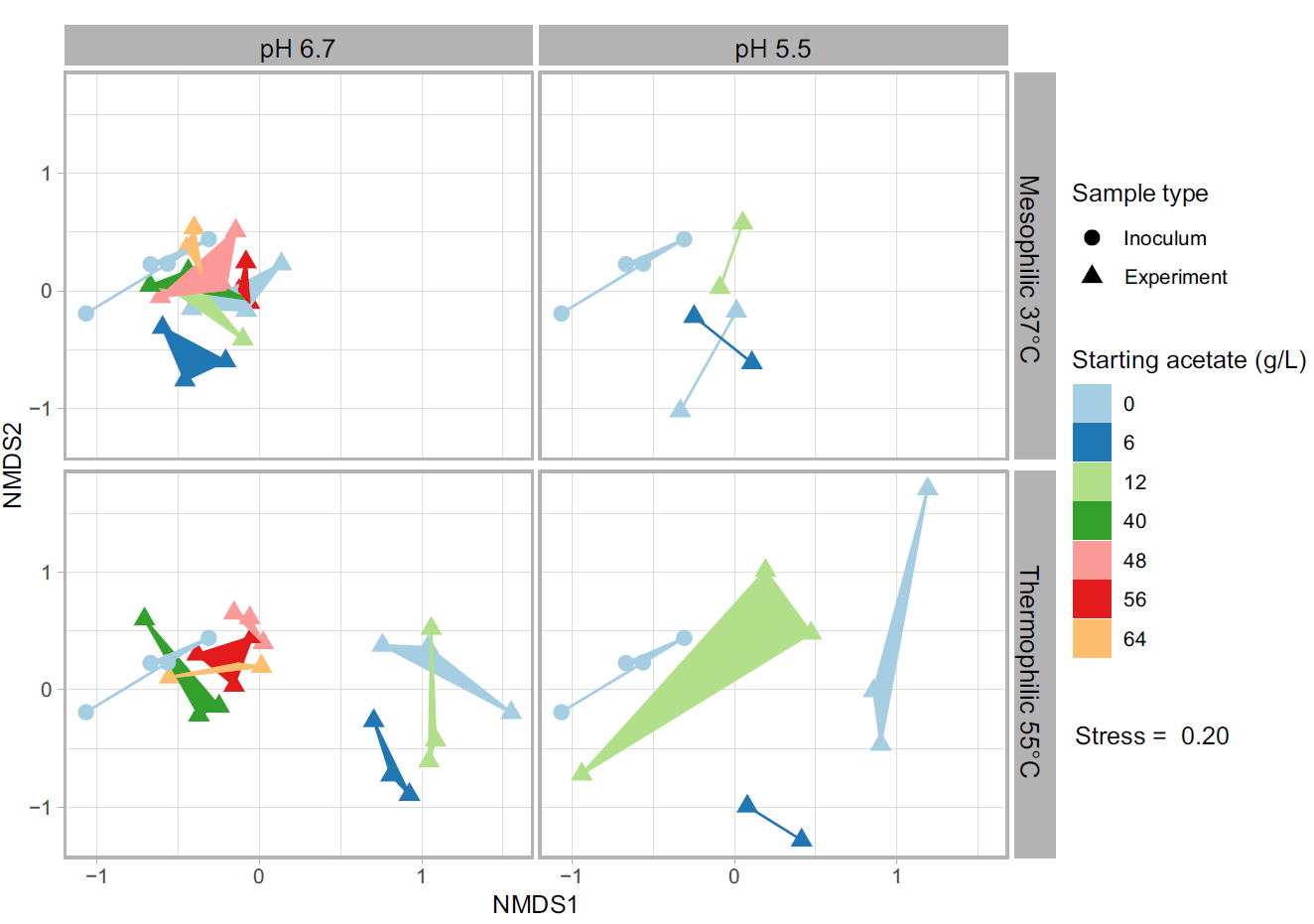


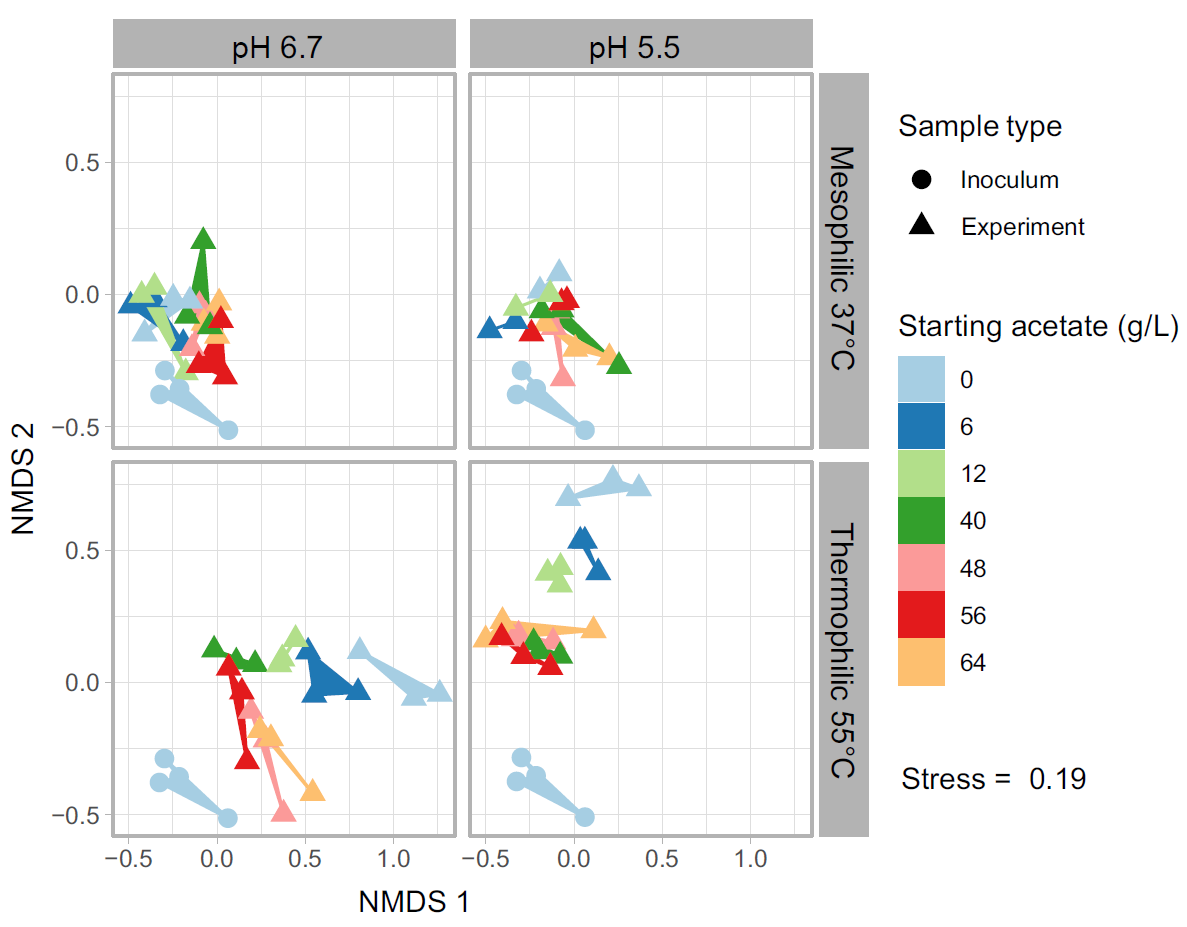
Figure S8. Non-metric multidimensional scaling (NMDS) plot based on the mcrA gene amplicon sequence variants (ASVs) representing the methanogenic share of the microbial community. Replicates are grouped by a polygon shape (triplicates) or a line (duplicates). The four inoculum samples were taken throughout a period of 6 months.

Figure S9. Non-metric multidimensional scaling (NMDS) plot based on the 16S rRNA gene amplicon sequence variants (ASVs) representing predominantly the bacterial share of the microbial community. Replicates are grouped by a polygon shape (triplicates) or a line (duplicates). The four inoculum samples were taken throughout a period of 6 months.





Figure S10. Consumption of CO over time. Error bars represent standard deviation among replicates (n=3).





Figure S11. Production of CH_4_ over time. Error bars represent standard deviation among replicates (n=3).





Figure S12. Production of H_2_ over time. Negative values indicate consumption. Error bars represent standard deviation among replicates (n=3).
